# The Essential and Optimal Analgesic and Anti-Inflammatory Medicines for Athletes at the Olympic Games

**DOI:** 10.1186/s40798-024-00743-3

**Published:** 2024-07-19

**Authors:** Mark Stuart, Mohammed Farooq, Trudy Thomas, Nada Mohamed-Ali, Mohammed Al-Maadheed, Vidya Mohamed-Ali

**Affiliations:** 1 International Testing Agency, Lausanne, Switzerland; 2 Centre for Metabolism and Inflammation, University College London, London, UK; 3 FIFA Medical Centre of Excellence, Orthopaedic and Sports Medicine Hospital, Aspetar, Doha, 29222 Qatar; 4 Medway School of Pharmacy, Universities of Kent and Greenwich, Chatham, UK; 5 Anti-Doping Lab Qatar, Sports City Road, Doha, Qatar

**Keywords:** Olympic, Pharmacy, Analgesics, Anti-inflammatory, Medicines, Pain, Athlete, Sports, Medicine, Formulary

## Abstract

**Background:**

In 2019, the International Olympic Committee published the first *Olympic and Paralympic Model Formulary* (OPF), which defined the standardised set of medications required at every Olympic and Paralympic Games for the treatment of athletes. This study aimed to test the OPF to determine whether it meets the clinical needs of the athlete population with respect to medications used for pain and/or inflammation (PI), and to present a revised set of essential PI medications for the OPF based on prevalence of athlete use. Medication-use data of athletes at the Tokyo 2020 and Beijing 2022 Olympic Games (*n* = 6155) from three sources were used to establish prevalence of PI medicine use and to revise the OPF: (i) doping control forms, (ii) pharmacy dispensing reports, and (iii) injection declaration forms. This revised list was further validated through (iv) medication importation declarations by teams (*n* = 156), and (v) survey of team physicians (*n* = 382).

**Results:**

Overall prevalence of PI medication use was 36.7%, with higher use by female athletes (female: 44.1%; male: 30.0%; *p* < 0.001), with non-steroidal anti-inflammatory drugs being the most used class (27%). Use of medications with safety risks were identified, including nimesulide, piroxicam and metamizole. A revised list of 48 PI medications was recommended for the OPF.

**Conclusion:**

The research led to a revised set of essential medications for the treatment of pain and inflammation to be available for athletes at the Olympic Games, which would lead to a 7% improvement in the numbers of athletes who could have their exact PI medication requirements met by the OPF.

**Supplementary Information:**

The online version contains supplementary material available at 10.1186/s40798-024-00743-3.

## Key Points

- Medicines are provided at the Olympic Games for prescribing to athletes by international team physicians, with those most commonly prescribed being for treatment of pain and inflammation due to musculoskeletal sports injury.
- This study presents an optimal list of 48 essential medicines for the treatment of pain and inflammation to be available at every summer and winter Olympic Games, selected based on prevalence of athlete use, avoidance of harms, and reflecting the preferred treatment options of international team physicians.
- The results of this study will inform the selection of medicines to be available through the medical services within the athlete villages and competition venues at future Olympic Games. The implementation of this formulary is expected to result in 7% more athletes having access to their preferred choice of treatment for pain and inflammation due to sports injury.

**Supplementary Information:**

The online version contains supplementary material available at 10.1186/s40798-024-00743-3.

## Background

At every Olympic Games, a medicines formulary is provided through the pharmacy in the athlete village and across the competition and training venues for the treatment of athletes. The medicines are chosen to meet the expected pharmacological needs of athletes in this unique sport setting for prescribing by both local, and team physicians accompanying each team. Prior to 2020, this formulary was developed on a games-by-games basis, and usually reflected the choice of medicines and national formulary of the host country. This led to significant variations between games in the types, standards, safety and costs of medicines provided. Despite medicines being available at the games, many teams have historically imported large stocks of their own medication for use at the games to guarantee they have their preferred treatments available if needed.

In 2019, the International Olympic Committee (IOC) published the first *Olympic and Paralympic Model Formulary* (OPF) [1], which determines a standardised set of medications required to be made available at every Olympic and Paralympic Games. The OPF was intended to provide athletes with a consistent, safe, and clinically relevant selection of medicines to access during the games, which represented the range of medications most commonly used by athletes. It was also intended to represent the prescribing preferences of the team physicians from all countries of the world, so as to reduce the need to import additional stock medications to each games [1]. 

Medications athletes take during the games for the treatment of illness or injury need to be recorded through several channels. Firstly, if a medication is dispensed to an athlete through the athlete village pharmacy, it is recorded in the dispensing reports. In addition, athletes must declare any medications they have taken in the previous seven days on the doping control form at the time of providing a sample for testing, as a requirement of the World Anti-Doping Code [2]. The IOC also requires all team physicians to declare the administration of injectable medications to athletes through the IOC Needle Policy, which is in place to ensure the safe and appropriate use of injections [3]. 

The use of medication to reduce pain and inflammation associated with training, competition or soft tissue injuries by athletes of all levels, has been widely reported [4, 5]. The use of non-steroidal anti-inflammatory drugs (NSAIDs) is significantly higher in Olympic athletes compared with age-matched controls [4, 6], High use of NSAIDs has been reported across all levels of athletes from college level through to Olympic level athletes across various athletics disciplines, with one study reporting that 50% of professional level football players use NSAIDs during the competition period [5, 7–12]. It is unsurprising that a significant portion of the OPF comprises medicines used for pain and inflammation and as such, this subset of medications is therefore ideal for the purposes of validation of the OPF in the Olympic setting.

The way analgesic and anti-inflammatory drugs are prescribed to athletes in the context of high-performance sport varies between clinicians, as does the intended use, which can range from immediate treatment of acute pain and inflammation, through to pain prevention, or performance continuation or improvement. In a survey of international team physicians on pain management conducted at the Rio 2016 Olympic Games, 61% (*n* = 107) of physicians reported routine prescribing of analgesics or anti-inflammatory medications in greater than 10% of athlete patients. In the same survey, 50% of physicians considered acute pain and inflammation to be the most important factor when prescribing analgesic and anti-inflammatory medications, with 7% considering prevention of pain, inflammation and delayed-onset muscle soreness to be important, and 6% considering performance or endurance improvement to be an important factor when prescribing analgesic and anti-inflammatory medications to elite athletes [13]. It is also widely reported in studies looking at drug use in elite athletes that because of their intense training and competition regimens, athletes are more likely to use multiple analgesic agents compared to the general population, and be using them prophylactically before competition for prevention of pain [14–16]. 

The OPF was implemented for the first time at the Tokyo 2020 Olympic Games and subsequently at the Beijing 2022 Olympic Winter Games. Analysis of records of actual medication use in a real-life setting at two international games covering all Olympic summer and winter sport disciplines could enable a comparison with the existing PI medications presented in the OPF, and an opportunity to revise the first edition of the OPF to better reflect prevalence of use among Olympic athletes.

The aim of this study was to test the OPF at a summer and winter Olympic Games to determine whether it met the clinical needs of the athlete population with respect to medications used for pain and/or inflammation (PI medications) of musculoskeletal conditions as a result of sport injuries in athletes, and to present a revised set of essential PI medications for the OPF based on prevalence of athlete use. The objectives were to:

1) Establish prevalence of PI medication use by athletes through direct evidence of use obtained through a review of medications declared by athletes on (i) doping control forms; (ii) pharmacy dispensing reports; (iii) injection declarations made by team physicians [3]. 

2) Compare the first edition of the OPF with the prevalence of PI medication use by athletes and revise it to reflect the medications in current use by athletes.

3) Validate the revised OPF with two further sources of indirect evidence of use: iv) declarations by team physicians of imported stock medication; and v) a cross-sectional survey of team physicians working at both Olympic and Paralympic Games on their prescribing choices for treatment of sports injury.

## Methods

The research was undertaken at the Tokyo 2020 Olympic Games, and the Beijing 2022 Olympic Winter Games. The study was fully inclusive of athletes of all genders, ethnicity, and race, with a diverse study population which included athletes, and their physicians, from 205 countries covering every winter and summer Olympic sport. The testing program for the Olympic Games, and the associated doping control form data, included an equal balance of male and female athletes; the authorship team was also gender balanced. In addition, the survey of team physicians used for validation purposes was also conducted at the Tokyo 2020 and Beijing 2022 Paralympic Games. The survey was available in multiple languages to allow for inclusivity.

“PI medications” were defined as those which could be used for either direct or adjunct treatment of pain and/or inflammation according to the therapeutic classification system used in the first OPF, which were: NSAIDs; COX-2 inhibitors; other non-opioids; opioids; medicines for neuropathic pain; local, and oral corticosteroids; local anaesthetics; benzodiazepines; skeletal muscle relaxants; massage and physical therapy preparations; and general anaesthetics with analgesia.

**Fig. 1:**
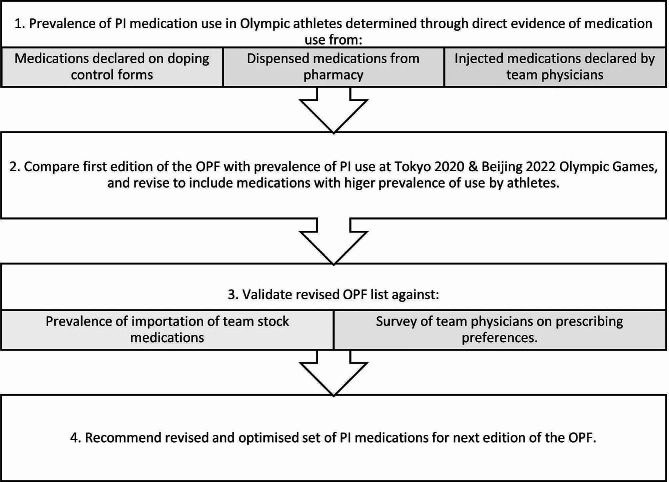
Schema of the study

The mixed methods study design is summarised in Fig. 1. It involved a retrospective review of reports of medication usage by athletes from the three data sources. Inclusion criteria were (i) athletes selected for testing through the doping control process; (ii) athletes dispensed a medication through the pharmacy in the athlete village; and (iii) athletes administered an injectable medication by their team physician. All data were provided as anonymised reports with all personal identifiers removed. All records of medication use were combined into one database, and any record of use that appeared in more than one of the datasets were removed, so as not to count a single use or administration of a medication more than once. Product names were translated into the generic medication name using *Martindale* [17]. 

### Inclusion Criteria for the Revised List of PI Medications

Medications were tabulated by therapeutic class and route of administration, against frequency and prevalence of use. Within each class and route category, the following rules were applied to revise the list of PI medications.

1. If any medication had a higher prevalence of use than any of the medications currently listed, it was added to the revised OPF.
2. If any medication on the original OPF was used by less than 0.2% of athletes, and a clinically interchangeable alternative in the same class and route was on the revised list, then it was omitted, *unless* it was indicated for treatment of an emergency or life-threatening condition.
3. Medications with known harms or safety risks, identified through any official warning by a medicines’ regulatory agency, were not recommended for inclusion, regardless of prevalence of use.

The cutoff value of 0.2% was selected based on an assessment of the resulting potential numbers of athletes who would be taking those medicines. At the Tokyo Olympics (*n* = 11,420), 0.2% represented 23 athletes, and at the Beijing Olympics (*n* = 2,834), 0.2% represented 6 athletes. These numbers of athletes were considered acceptable in terms of not being able to provide an exact match of their current medications on the revised OPF, as a many of these medications were also available via team stock. Or if they were not, then access to non-formulary medicines is always possible through the medical services at the Olympic Games, if required. For such low numbers, the burden on pharmacy services to access such drugs if absolutely required would be minimal, and would be acceptable based on the historical level of access for non-formulary drug at previous games; at the London 2012 Olympic and Paralympic Games, 8% of all drugs required were not on the formulary, which was not problematic [18]. 

#### National Olympic Committee (NOC) Team Stock Medicines Importation Declaration Review

The resulting revised list of PI medications was further validated through a retrospective review of declarations of all NOC team stock medication imported to the Tokyo or Beijing Olympic Games as required by the customs and import laws. Total counts of imported PI medication types, therapeutic categories, routes of administration and country of the importing teams were tabulated. The results were compared with the revised set of PI medications to determine to what extent the revised list of PI medications matched medications carried as stock by teams.

#### Survey of NOC Team Physicians

An additional validation of the revised OPF was undertaken through an online cross-sectional survey of team physicians at the Tokyo 2020 and Beijing 2022 Olympic and Paralympic Games, with the inclusion criterion as any prescribing physician attending one or more of these games. Every team physician was invited to complete the survey through announcements made at the pre-games team physician meetings held before the opening ceremony of each games. The survey comprised of 25 categorical, scaled, and open-ended questions, which aimed to evaluate the prescribing preferences of physicians relating to analgesic and anti-inflammatory medicines for treatment of musculoskeletal injury in athletes. The survey was hosted on Google Forms and available in English, French, Spanish, Russian and Japanese, and respondents could select their preferred language to complete the survey. The results of the questions relating to the prescribing preferences of team physicians were compared with the revised list of PI medications to assess the extent to which their preferences could be met.

Statistical analyses. All data were analysed using SPSS (v21, USA) [19], with frequencies and percentages used to describe categorical variables including: event, medication, therapeutic class, and route of administration. The chi-square test of independence was performed to compare the differences between these variables. A *p*-value < 0.05 was considered the cut-off for statistical significance.

This research was reviewed by the University College London (UCL) Research Ethics Committee (reference 18,955/001) and registered as health research with the UCL Data Protection Office (reference Z6364106/2020/09/14) in line with UCL’s Data Protection Policy.

## Results

Records of medication use were obtained for a total of 6,155 of 14,254 (43.2%) athletes across the Tokyo 2020 and Beijing 2022 Olympic Games (summer: *n* = 4,492 (39.3%); winter: *n* = 1,663 (58.7%)). The study population included athletes from 197 of 205 (96%) countries, and all 40 Olympic winter and summer sports [20]. 

### Prevalence of PI Medication Use

The overall prevalence of PI medication use was 36.7%, but prevalence varied between the summer and winter Olympic Games. In Beijing, 487 of 1663 athletes (29.3%) used PI medications. In Tokyo, the prevalence was significantly higher, with 1771 of 4492 athletes (39.4%) using PI medications (*p* < 0.001). The prevalence of individual medication use varied between different OPF classes and routes of administration (Table 1). Overall, oral NSAIDs was the most used medication, with 25.6% of athletes using them, followed by other oral non-opioid analgesics with a prevalence of 9.8%.

**Table 1:** Prevalence of PI medication use by Tokyo 2020 & Beijing 2022 Olympic athletes per OPF class and route

OPF Class	Route of Administration	Number of Athletes (*n* = 6155)	Prevalence
01.1.1 NSAID	Injection	62	1.01%
	Oral	1578	25.64%
	Rectal	5	0.08%
	Topical	121	1.97%
	Transdermal	49	0.80%
01.1.2 COX-2 inhibitor	Injection	1	0.02%
	Oral	172	2.79%
01.1.3 Other non-opioid analgesic	Injection	1	0.02%
	Oral	603	9.80%
	Rectal	1	0.02%
01.2 Opioid analgesics	Injection	7	0.11%
	Intranasal	1	0.02%
	Oral	59	0.96%
	Transdermal	1	0.02%
01.3 Medicines for neuropathic pain	Oral	16	0.26%
01.4 Corticosteroids for local injection	Injection	103	1.67%
01.5 Local anaesthetics	Injection	144	2.34%
	Topical	23	0.37%
	Transdermal	5	0.08%
01.5.1 Local anaesthetics + vasoconstrictor	Injection	7	0.11%
02.1 Corticosteroids for oral use	Oral	7	0.11%
03.1 Benzodiazepines	Injection	2	0.03%
	Oral	101	1.64%
03.2 Skeletal muscle relaxants	Injection	6	0.10%
	Oral	76	1.23%
	Topical	1	0.02%
10.9 Massage and physical therapy preparations	Topical	10	0.16%
	Transdermal	1	0.02%
23.1.2 General anaesthetics with analgesia	Inhalation	1	0.02%
	Injection	1	0.02%

Overall, female athletes had a higher prevalence of PI medication use compared to male athletes (female: 44.1%; male: 30.0%; *p* < 0.001). Notable differences between male and female prevalence were observed in some therapeutical categories, including a higher prevalence of NSAID use in female athletes (female: 33.4%; male: 26.0%; *p* < 0.001), and higher use of other non-opioid analgesics in females compared to males (female: 13.3%; male: 7.8%; *p* < 0.001).

No significant difference was observed in the proportion of athletes taking medications within each PI therapeutic classes between Tokyo and Beijing, except for local corticosteroid injections which had a higher proportion of use in Tokyo (3.5%), compared to Beijing (1.4%; *p* < 0.05) (Table 2).

**Table 2:** Records of PI medication use by Olympic athletes at Beijing 2022 and Tokyo 2020 per OPF class

OPF Class	Beijing PI use records	Tokyo PI use records
01.1.1 NSAID	440 (63.1%)	1630 (59.8%)
01.1.2 COX-2 inhibitor	27 (3.9%)	144 (5.3%)
01.1.3 Non-opioid analgesic	129 (18.5%)	475 (17.4%)
01.2 Opioid analgesics	14 (2.0%)	53 (1.9%)
01.3 Medicines for neuropathic pain	3 (0.4%)	12 (0.4%)
01.4 Corticosteroids for local injection	10 (1.4%)	95 (3.5%)*
01.5 Local anaesthetics	32 (4.6%)	153 (5.6%)
01.5.1 Local anaesthetics + vasoconstrictor	3 (0.4%)	3 (0.1%)
02.1 Corticosteroids for oral use	1 (0.1%)	5 (0.2%)
03.1 Benzodiazepines	24 (3.4%)	81 (3.0%)
03.2 Skeletal muscle relaxants	12 (1.7%)	68 (2.5%)
10.9 Massage and physical therapy preparations	2 (0.3%)	7 (0.3%)
23.1.2 General anaesthetics with analgesia	0 (0.0%)^a^	0 (0.0%)^a^
**Total unique records**	**697 (100%)**	**2726 (100%)**

*P < 0.05; ^a^ Not used in the comparisons

### The Revised List of PI Medications

The revision of the first edition of the OPF resulted in a new list of 48 PI medications (Table 3). There were 9 new medications added (shown in bold, with*), and 13 recommended for deletion due to either low or no evidence of use, or documented safety risks (Table 4). If implemented in the same population, this revised list of PI medications would lead to a 7% improvement in numbers of athletes who could have their exact PI medication requirements met by the OPF.

**Table 3:** PI medications for inclusion in the revised OPF

OPF Class	Route	Drug
01.1.1 NSAID	Injection	Diclofenac
		Ketorolac
	Oral	Aspirin
		Diclofenac
		Ibuprofen
		**Ketoprofen** ^*^
		Ketorolac
		Loxoprofen
		Meloxicam
		Naproxen
	Rectal	Diclofenac
	Topical	Diclofenac
		Ibuprofen
	Transdermal	**Diclofenac** ^*^
01.1.2 COX-2 inhibitor	Oral	Celecoxib
		**Etoricoxib** ^*^
01.1.3 Non-opioid analgesic	Oral	Paracetamol
01.2 Opioid analgesics	Injection	Fentanyl
		Morphine
		Tramadol
	Oral	Codeine
		Codeine + Paracetamol
		Tramadol
01.3 Medicines for neuropathic pain	Oral	Duloxetine
		Gabapentin
		**Amitriptyline** ^*^
01.4 Corticosteroids for intra-articular use	Injection	**Betamethasone** ^*^
		Dexamethasone
		Triamcinolone
01.5 Local anaesthetics	Injection	Lidocaine
	Topical	Lidocaine
	Transdermal	Lidocaine
01.5.1 Local anaesthetics + vasoconstrictor	Injection	Lidocaine + Epinephrine
02.1 Corticosteroids for oral use	Oral	Prednisolone
03.1 Benzodiazepines	Injection	Diazepam
	Injection	Midazolam
	Oral	**Alprazolam** ^*^
		**Brotizolam** ^*^
		Diazepam
		Lorazepam
	Rectal	Diazepam
03.2 Skeletal muscle relaxants	Oral	**Chlorzoxazone** ^*^
		Tizanidine
10.9 Massage and physical therapy preps	Topical	**Capsaicin** ^*^
		Menthol
23.1.2 General anaesthetics with analgesia	Inhalation	Methoxyflurane
		Nitrous oxide + Oxygen
	Injection	Ketamine

^*^Newly added preparations

**Table 4:** PI medications deleted from the original OPF

OPF Class	Route	Drug
01.1.3 Non-opioid analgesic	Rectal	Paracetamol
01.2 Opioid analgesics	Oral	Dihydrocodeine
		Hydromorphone
01.3 Medicines for neuropathic pain	Oral	Nortriptyline
		Pregabalin
01.4 Corticosteroids for intra-articular use	Injection	Hydrocortisone
01.5.1 Local anaesthetics + vasoconstrictor	Injection	Bupivacaine + Epinephrine
02.1 Corticosteroids for oral use	Oral	Beclometasone
		Dexamethasone
03.1 Benzodiazepines	Injection	Lorazepam
	Oral	Midazolam
03.2 Skeletal muscle relaxants	Oral	Baclofen
10.9 Massage and physical therapy preps	Topical	Methylsalicylate + Menthol

### Validating the Revised List of PI Medications

#### NOC Team Stock Medicines Importation Declaration Review

Over both the Tokyo 2020 and Beijing 2022 Olympic games, a total of 156 of 205 (76.1%) teams imported stock medications for use at the games. Of all unique declarations, there were 1747 declarations for PI medications (Tokyo: 1248 (71%); Beijing: 499 (29%)). Online Supplementary Material (Table S1) shows the results, with prevalence of importation for all PI medications. The comparisons of imported PI medications with the revised list of PI medications are also provided in the Online Supplementary Material (Figures S1-S19). In comparing the revised list of PI medications with the prevalence of imported team stock medicines, it was found to include the most frequently stocked PI medications in 17 of 18 (94%) of all PI therapeutic categories.

#### Survey of NOC Team Physicians

A total of 382 team physicians from 120 different countries completed the survey. There were 279 respondents from the Tokyo 2020 Olympic and Paralympic Games (Olympics: *n* = 200; Paralympics: *n* = 79), and 103 respondents from the Beijing 2022 Olympic and Paralympic Winter Games (Olympics: *n* = 87; Paralympics: *n* = 16).

The majority of physicians (66%, *n* = 250) expected up to 30% of athletes to require analgesic or anti-inflammatory medication during the games. A smaller proportion (21.6%, *n* = 82) expected 31–60% of athletes to require these medications, while even less (12.4%, *n* = 47) expected 61–100% of athletes to require these types of medications (Online Supplementary Material, Figure S25). The survey asked what proportion of their expected PI medications for sports-related conditions could be covered by the medicines in the games formulary, with 73% (*n* = 277) reporting either most (> 75%) or all of their PI prescriptions could be covered by the existing formulary. Only 16.7% (*n* = 63) indicated that less than 25% of their prescriptions could be covered (Online Supplementary Material, Figure S26).

Team physicians were also asked which oral non-opioid or NSAIDs they had prescribed in the last 12 months for sports-related conditions in athletes. All physicians reported they had prescribed paracetamol (100%, *n* = 381), with diclofenac (78.2%, *n* = 298) and ibuprofen (77.4%, *n* = 295) also being used by the majority of respondents. Naproxen (42.3%, *n* = 161), celecoxib (36.7%, *n* = 140), and meloxicam (25.7%, *n* = 98) were also commonly used. Other oral drugs in this category were used by less than 25% of physicians in the previous 12 months. The survey also asked which oral opioid analgesic drugs that team physicians had prescribed for sports-related conditions in athletes in the previous 12 months. The most commonly prescribed drugs were oral tramadol (40.8%, *n* = 122) and oral codeine (26.4%, *n* = 79). Other oral drugs in this class were prescribed by less than 6% of physicians.

The survey continued to asked the preferred oral, injectable or topical medications that team physicians prescribe for a comprehensive range of clinical indications relating to musculoskeletal injury in sport. These included for mild, moderate and severe pain, with or without inflammation; for neuropathic pain; as a muscle relaxant; for emergency analgesia of the field of play; and for rapid-onset analgesia with sedation on the field of play. The results were then compared to the set of medications proposed in the revised OPF. The full results of the survey and the comparison of the most frequently prescribed medications, with the recommended revised list for the OPF are provided in the Online Supplementary Material, Appendix 2 (Figures S20-S44). The survey showed that in every therapeutic category of the revised OPF, the medication example most frequently prescribed by the team physicians was now listed.

## Discussion

The three sources of medication use data used covered an extensive cross-sample of athletes at both winter and summer Olympic games (*n* = 6155), being sufficiently large enough to be representative of the wider population of athletes from every sport, and most participating countries to give a reliable estimate of prevalence. The model used to determine the inclusion of medications for each separate OPF class and route subset, was effective in recommending substitutions to medication examples within each class with a higher prevalence of use. The data were also effective in highlighting medications rarely or never used, which effectively informed the rationalisation of the OPF to a pragmatic and likely to be used set of treatment options. But the rules also ensured that drugs for emergency use, which are rarely used but must be available, such as morphine or methoxyflurane, were retained despite few or no records of use.

The consistencies in the prevalence of use for the various PI medication classes between both summer and winter games, confirm that a single unified formulary pertaining to both the summer and winter games is appropriate, rather than different versions for each event. The only difference was the use of corticosteroids by local injection, which showed a significantly higher prevalence of use in Tokyo than Beijing. However, the lower use in Beijing can be explained by the prior prohibition of local injections of corticosteroids by the World Anti-Doping Agency in January 2022; likely resulting in physicians opting for other permitted alternatives.

The study also showed a higher overall prevalence of PI medication use among female (44.1%) compared to male athletes (30.0%; *p* < 0.001), and most notably a higher prevalence of NSAID use in female (33.4%) compared to male athletes (26.0%; *p* < 0.001). However, these findings are not unique to the sport setting, with other studies in the general population describing higher analgesic use among females [21, 22]. These findings are interesting to compare with the IOC Injury and Illness Study, which reported no significant difference in overall injury incidence between male and female athletes at both the Tokyo 2020 and Beijing 2022 Olympic Games [23, 24]. This disparity in PI medication use between male and female athletes, despite similar injury rates may be due to comorbidities, such as dysmenorrhoea in female athletes, but warrants further investigation to fully understand the underlying reasons.

### Validating the Revised List against the Team Stock Importation Review

With the revised OPF list containing the most frequently imported PI medications in all but one of the PI therapeutic categories, the results suggest the revised list of PI medications is well-suited to meet the anticipated prescribing needs of the team physicians. This validation provided additional evidence that the approach to revising the OPF delivered a list of medications broadly aligned to what teams also carry as stock. Improving the OPF to better match what teams carry to the games, may result in a reduced need for teams to import their own stock, with greater utilisation of local supplies.

### Validating the Revised List against the Survey of Team Physicians

The team physician survey explored the most preferred and frequently prescribed medications for various sports-related conditions in athletes. Overall, the findings demonstrate that the revised list of PI medications is well aligned with the prescribing preferences of team physicians. For every medication category questioned by the survey, every one, or more, of the top most prescribed PI medications were included on the revised list, providing reassurance that the revised list can effectively address the preferences of team physicians in relation to treatment of pain and inflammation in athletes.

### Limitations

While providing insights into medication use among athletes through multiple sources, this study presents some limitations. The accuracy of doping control form data is dependent on athletes’ honesty, capturing only a 7-day period of use, which may not represent chronic or longer-term treatments. The selection bias towards athletes tested for doping, potential under-reporting or intentional misinformation potentially constrain the validity of these findings. The pharmacy data, although reliable, is not fully representative of all countries due to varying access by different teams. Lastly, the rate of compliance to the IOC Needle Policy is unknown [25], and so injection declarations may potentially be under-reported. The data used to validate the revised PI medication list, imported team stock, and the prescribing preferences of team physicians reported through the survey, do not necessarily reflect actual medication use, but rather anticipated or expected usage. Despite these limitations, the study offers the most comprehensive assessment of prevalence of medication use possible in the Olympic Games setting according to available data.

It must be noted that this study considered the prevalence of medication use in Olympic athletes only. However, the OPF is intended to provide a common set of medications for both Olympic and Paralympic Games. As such, further analysis of the prevalence of medications use in Paralympic athletes is warranted to ensure full applicability of any subsequent edition of the OPF to both settings. The survey of team physicians presented was also undertaken at the Paralympic Games, which provides initial validation that the revised list is likely to also be appropriate for the Paralympic setting.

### Clinical Implications

This study ensures that the medications selected to be included in future editions of the OPF will be more relevant to the athlete population in this setting, enabling a greater number of athletes and their prescribers to have their exact treatment choice available. It also ensures that any medication offered has been considered with respect to avoidance of harm and the protection of athletes health. This study resulted in the identification and exclusion of medications in current use with known potential safety risks to athletes, including nimesulide, piroxicam and metamizole [26–28]. The quantitative approach to revising the OPF can also be repeated periodically after future games to ensure that the OPF is continually updated and evolves to reflect the current needs of athletes.

A games formulary aims to provide the both the complete medicine needs for athletes while at the games, and to cover the range of medications that team physicians prescribe to their athletes so they do not have to import their own stocks to the games. By providing a selection of drugs that meets the majority of clinical needs and prescribing choice, the shift in reliance on the medicines of the host country compared to imported medicines can be slowly supported. Reducing the need to import medicines to each games will inevitably lead to reduced cost to teams, reduce wastage and a significant reduction of the carbon footprint that results in the transport of multiple large stocks of medicines to the host country.

## Conclusion

Through this study, the first edition of the OPF was revised using a quantitative, prevalence-based approach to better reflect the actual needs of Olympic athletes, with the results used to inform subsequent editions of the OPF. Use of several medicines with known potential harms, but still being used by athletes, were identified which warrants further education within the sports community to caution against their use. Further analysis of PI medication use in Paralympic athletes is also recommended to ensure the applicability of the revised OPF to the athletes of the Paralympic Games.

Ultimately, the revised OPF will provide athletes with access to the medications they require during the Olympic Games, reduce the risks to health from harmful drugs, while reducing the burden on teams to import their own medication stock.

### Electronic Supplementary Material

Below is the link to the electronic supplementary material.

Supplementary Material 1

## Data Availability

All results data relevant to the study are included in the article. The raw data related to each area of the study are not publicly available to protect the privacy of the participants.

## Abbreviations

IOC: International Olympic Committee
IPC: International Paralympic Committee
OPF: Olympic and Paralympic Model Formulary
PI: Pain and Inflammation
