# Supplementary material for: The Essential and Optimal Analgesic and Anti-Inflammatory Medicines for Athletes at the Olympic Games
Source: Sports Med Open. 2024 Jul 19;10:80. doi: 10.1186/s40798-024-00743-3 (PMC11258109; doi:10.1186/s40798-024-00743-3)
Supplement: Supplementary file 1 — Supplementary Material 1 [file 40798_2024_743_MOESM1_ESM.docx]

**The essential and optimal analgesic and anti-inflammatory medicines for athletes at the Olympic Games. *Sports Medicine*.**

**ONLINE SUPPLEMENTARY MATERIAL**

**Authors**

Dr Mark Stuart^1,2^ [Corresponding author: mark.stuart.18@ucl.ac.uk]

Dr Mohammed Farooq^3^

Dr Trudy Thomas^4^

Nada Mohamed-Ali^2^

Prof Mohammed Al-Maadheed^2,5^

Prof Vidya Mohamed-Ali^2,5^

**Affiliations**

1. International Testing Agency, Lausanne, Switzerland
2. Centre for Metabolism and Inflammation, University College London, United Kingdom
3. Aspetar, FIFA Medical Centre of Excellence, Orthopaedic and Sports Medicine Hospital, Doha 29222, Qatar
4. Medway School of Pharmacy, Universities of Kent and Greenwich, Chatham, United Kingdom
5. Anti-Doping Lab Qatar, Sports City Road, Doha, Qatar

**Appendix 1. Results of the NOC team stock medicines importation declaration review**

The NOC team drug importation declarations across the Tokyo 2020 and Beijing 2022 Olympic Games are tabulated below.

Table S1. NOC team importation declarations by frequency, % of drugs within each OPF class and route category, and prevalence of team importation.

| **OPF Class** | **Route** | **Drug** | **Countries importing (n=156)** | **n%** | **Prevalence** |
| --- | --- | --- | --- | --- | --- |
| **01.1.1 NSAID** | **Injection** | Diclofenac | 45 | 41.7% | 29% |
|  |  | Ketorolac | 18 | 16.7% | 12% |
|  |  | Ketoprofen | 17 | 15.7% | 11% |
|  |  | Piroxicam | 9 | 8.3% | 6% |
|  |  | Dexketoprofen | 8 | 7.4% | 5% |
|  |  | Meloxicam | 7 | 6.5% | 4% |
|  |  | Aspirin | 3 | 2.8% | 2% |
|  |  | Lornoxicam | 1 | .9% | 1% |
|  |  | Loxoprofen | 0 | 0.0% | 0% |
|  |  | Total | 108 | 100.0% |  |
|  | **Oral** | Diclofenac | 82 | 16.7% | 53% |
|  |  | Ibuprofen | 77 | 15.7% | 49% |
|  |  | Aspirin | 72 | 14.6% | 46% |
|  |  | Naproxen | 41 | 8.3% | 26% |
|  |  | Meloxicam | 29 | 5.9% | 19% |
|  |  | Ketoprofen | 25 | 5.1% | 16% |
|  |  | Nimesulide | 23 | 4.7% | 15% |
|  |  | Ketorolac | 17 | 3.5% | 11% |
|  |  | Mefenamic acid | 15 | 3.0% | 10% |
|  |  | Dexketoprofen | 14 | 2.8% | 9% |
|  |  | Aceclofenac | 12 | 2.4% | 8% |
|  |  | Piroxicam | 12 | 2.4% | 8% |
|  |  | Indomethacin | 10 | 2.0% | 6% |
|  |  | Flurbiprofen | 8 | 1.6% | 5% |
|  |  | Lornoxicam | 8 | 1.6% | 5% |
|  |  | Aspirin + Paracetamol | 7 | 1.4% | 4% |
|  |  | Naproxen + Esomeprazole | 5 | 1.0% | 3% |
|  |  | Dexibuprofen | 4 | .8% | 3% |
|  |  | Diclofenac + Misoprostol | 4 | .8% | 3% |
|  |  | Tenoxicam | 4 | .8% | 3% |
|  |  | Acemetacin | 3 | .6% | 2% |
|  |  | Ibuprofen + Paracetamol | 3 | .6% | 2% |
|  |  | Loxoprofen | 3 | .6% | 2% |
|  |  | Propyphenazone + Paracetamol | 3 | .6% | 2% |
|  |  | Tiaprofenic acid | 2 | .4% | 1% |
|  |  | Aceclofenac + Paracetamol | 1 | .2% | 1% |
|  |  | Diclofenac + Carisoprodol | 1 | .2% | 1% |
|  |  | Diclofenac + Omeprazole | 1 | .2% | 1% |
|  |  | Diclofenac + Paracetamol | 1 | .2% | 1% |
|  |  | Diclofenac + Paracetamol + Carisoprodol | 1 | .2% | 1% |
|  |  | Etodolac | 1 | .2% | 1% |
|  |  | Ketoprofen + Paracetamol | 1 | .2% | 1% |
|  |  | Ketoprofen + Paracetamol + Carisoprodol | 1 | .2% | 1% |
|  |  | Niflumic acid | 1 | .2% | 1% |
|  |  | Aminophenazone | 0 | 0.0% | 0% |
|  |  | Analgesic (non-descript) | 0 | 0.0% | 0% |
|  |  | Antiinflammatory (non-descript) | 0 | 0.0% | 0% |
|  |  | Nabumatone | 0 | 0.0% | 0% |
|  |  | NSAID (non-descript) | 0 | 0.0% | 0% |
|  |  | Total | 492 | 100.0% |  |
|  | **Rectal** | Diclofenac | 14 | 66.7% | 9% |
|  |  | Indomethacin | 3 | 14.3% | 2% |
|  |  | Piroxicam | 2 | 9.5% | 1% |
|  |  | Ketoprofen | 1 | 4.8% | 1% |
|  |  | Nimesulide | 1 | 4.8% | 1% |
|  |  | Total | 21 | 100.0% |  |
|  | **Topical** | Diclofenac | 75 | 53.2% | 48% |
|  |  | Ketoprofen | 22 | 15.6% | 14% |
|  |  | Ibuprofen | 11 | 7.8% | 7% |
|  |  | Etofenamate | 5 | 3.5% | 3% |
|  |  | Flurbiprofen | 5 | 3.5% | 3% |
|  |  | Diethylamine salicylate | 4 | 2.8% | 3% |
|  |  | Indomethacin | 4 | 2.8% | 3% |
|  |  | Piroxicam | 4 | 2.8% | 3% |
|  |  | Dexketoprofen | 2 | 1.4% | 1% |
|  |  | Nimesulide | 2 | 1.4% | 1% |
|  |  | Phenylbutazone | 2 | 1.4% | 1% |
|  |  | Aceclofenac | 1 | .7% | 1% |
|  |  | Etodolac | 1 | .7% | 1% |
|  |  | Hydroxyethyl salicylate | 1 | .7% | 1% |
|  |  | Ibuprofen + Menthol | 1 | .7% | 1% |
|  |  | Ketorolac | 1 | .7% | 1% |
|  |  | Loxoprofen | 0 | 0.0% | 0% |
|  |  | Naproxen | 0 | 0.0% | 0% |
|  |  | Total | 141 | 100.0% |  |
|  | **Transdermal** | Diclofenac | 24 | 68.6% | 15% |
|  |  | Ketoprofen | 5 | 14.3% | 3% |
|  |  | Flurbiprofen | 4 | 11.4% | 3% |
|  |  | Loxoprofen | 1 | 2.9% | 1% |
|  |  | Piroxicam | 1 | 2.9% | 1% |
|  |  | Total | 35 | 100.0% |  |
| **01.1.2 COX-2 inhibitor** | **Injection** | Parecoxib | 4 | 100.0% | 3% |
|  |  | Total | 4 | 100.0% | 3% |
|  | **Oral** | Etoricoxib | 38 | 57.6% | 24% |
|  |  | Celecoxib | 27 | 40.9% | 17% |
|  |  | Rofecoxib | 1 | 1.5% | 1% |
|  |  | Parecoxib | 0 | 0.0% | 0% |
|  |  | Total | 66 | 100.0% |  |
| **01.1.3 Non-opioid analgesic** | **Injection** | Metamizole | 25 | 86.2% | 16% |
|  |  | Paracetamol | 3 | 10.3% | 2% |
|  |  | Nefopam | 1 | 3.4% | 1% |
|  |  | Total | 29 | 100.0% |  |
|  | **Oral** | Paracetamol | 94 | 77.7% | 60% |
|  |  | Metamizole | 27 | 22.3% | 17% |
|  |  | Total | 121 | 100.0% |  |
|  | **Rectal** | Paracetamol | 5 | 100.0% | 3% |
|  |  | Total | 5 | 100.0% |  |
| **01.2 Opioid analgesics** | **Injection** | Tramadol | 10 | 66.7% | 6% |
|  |  | Morphine | 4 | 26.7% | 3% |
|  |  | Naloxone | 1 | 6.7% | 1% |
|  |  | Fentanyl | 0 | 0.0% | 0% |
|  |  | Total | 15 | 100.0% |  |
|  | **Intranasal** | Fentanyl | 1 | 100.0% | 1% |
|  |  | Total | 1 | 100.0% |  |
|  | **Oral** | Tramadol | 28 | 45.2% | 18% |
|  |  | Tramadol + Paracetamol | 11 | 17.7% | 7% |
|  |  | Codeine | 9 | 14.5% | 6% |
|  |  | Codeine + Paracetamol | 7 | 11.3% | 4% |
|  |  | Dihydrocodeine | 3 | 4.8% | 2% |
|  |  | Codeine + Diclofenac | 1 | 1.6% | 1% |
|  |  | Morphine | 1 | 1.6% | 1% |
|  |  | Tilidine | 1 | 1.6% | 1% |
|  |  | Tilidine + Naloxone | 1 | 1.6% | 1% |
|  |  | Codeine + Paracetamol + Aspirin | 0 | 0.0% | 0% |
|  |  | Codeine + Paracetamol + Ibuprofen | 0 | 0.0% | 0% |
|  |  | Dihydrocodeine + Paracetamol | 0 | 0.0% | 0% |
|  |  | Tramadol + Dexketoprofen | 0 | 0.0% | 0% |
|  |  | Total | 62 | 100.0% |  |
|  | **Transdermal** | Fentanyl | 0 | 0.0% | 0% |
|  |  | Total | 0 | 0.0% |  |
| **01.3 Medicines for neuropathic pain** | **Oral** | Gabapentin | 3 | 60.0% | 2% |
|  |  | Amitriptyline | 2 | 40.0% | 1% |
|  |  | Duloxetine | 0 | 0.0% | 0% |
|  |  | Nortriptyline | 0 | 0.0% | 0% |
|  |  | Pregabalin | 0 | 0.0% | 0% |
|  |  | Total | 5 | 100.0% |  |
| **01.4 Corticosteroids for intra-articular use** | **Injection** | Dexamethasone | 30 | 21.7% | 19% |
|  |  | Triamcinolone | 30 | 21.7% | 19% |
|  |  | Betamethasone | 28 | 20.3% | 18% |
|  |  | Methylprednisolone | 26 | 18.8% | 17% |
|  |  | Hydrocortisone | 12 | 8.7% | 8% |
|  |  | Prednisolone | 10 | 7.2% | 6% |
|  |  | Methylprednisolone + Benzocaine | 1 | .7% | 1% |
|  |  | Methylprednisolone + Lidocaine | 1 | .7% | 1% |
|  |  | Cortisone | 0 | 0.0% | 0% |
|  |  | Total | 138 | 100.0% |  |
| **01.5 Local anaesthetics** | **Injection** | Lidocaine | 61 | 58.1% | 39% |
|  |  | Bupivacaine | 23 | 21.9% | 15% |
|  |  | Procaine | 8 | 7.6% | 5% |
|  |  | Ropivacaine | 8 | 7.6% | 5% |
|  |  | Mepivacaine | 4 | 3.8% | 3% |
|  |  | Levobupivacaine | 1 | 1.0% | 1% |
|  |  | Anaesthetic (non-descript) | 0 | 0.0% | 0% |
|  |  | Mesocaine | 0 | 0.0% | 0% |
|  |  | Prilocaine | 0 | 0.0% | 0% |
|  |  | Trimecaine | 0 | 0.0% | 0% |
|  |  | Total | 105 | 100.0% |  |
|  | **Topical** | Lidocaine | 28 | 62.2% | 18% |
|  |  | Lidocaine + Prilocaine | 11 | 24.4% | 7% |
|  |  | Benzocaine | 4 | 8.9% | 3% |
|  |  | Dibucaine | 1 | 2.2% | 1% |
|  |  | Procaine | 1 | 2.2% | 1% |
|  |  | Articaine | 0 | 0.0% | 0% |
|  |  | Lidocaine + Chlorhexidine | 0 | 0.0% | 0% |
|  |  | Total | 45 | 100.0% |  |
|  | **Transdermal** | Lidocaine | 14 | 77.8% | 9% |
|  |  | Lidocaine + Menthol | 2 | 11.1% | 1% |
|  |  | Lidocaine + Prilocaine | 2 | 11.1% | 1% |
|  |  | Total | 18 | 100.0% |  |
| **01.5.1 Local anaesthetics + vasoconstrictor** | **Injection** | Lidocaine + Adrenaline | 1 | 100.0% | 1% |
|  |  | Lidocaine + Epinephrine | 0 | 0.0% | 0% |
|  |  | Total | 1 | 100.0% |  |
| **02.1 Corticosteroids for oral use** | **Oral** | Prednisolone | 28 | 56.0% | 18% |
|  |  | Methylprednisolone | 10 | 20.0% | 6% |
|  |  | Dexamethasone | 7 | 14.0% | 4% |
|  |  | Prednisone | 3 | 6.0% | 2% |
|  |  | Betamethasone | 1 | 2.0% | 1% |
|  |  | Triamcinolone | 1 | 2.0% | 1% |
|  |  | Fludrocortisone | 0 | 0.0% | 0% |
|  |  | Total | 50 | 100.0% |  |
| **03.1 Benzodiazepines** | **Injection** | Diazepam | 6 | 66.7% | 4% |
|  |  | Midazolam | 3 | 33.3% | 2% |
|  |  | Total | 9 | 100.0% |  |
|  | **Oral** | Diazepam | 14 | 41.2% | 9% |
|  |  | Bromazepam | 6 | 17.6% | 4% |
|  |  | Lorazepam | 3 | 8.8% | 2% |
|  |  | Alprazolam | 2 | 5.9% | 1% |
|  |  | Midazolam | 2 | 5.9% | 1% |
|  |  | Oxazepam | 2 | 5.9% | 1% |
|  |  | Brotizolam | 1 | 2.9% | 1% |
|  |  | Clonazepam | 1 | 2.9% | 1% |
|  |  | Etizolam | 1 | 2.9% | 1% |
|  |  | Lormetazepam | 1 | 2.9% | 1% |
|  |  | Triazolam | 1 | 2.9% | 1% |
|  |  | Cinolazepam | 0 | 0.0% | 0% |
|  |  | Estazolam | 0 | 0.0% | 0% |
|  |  | Phenazepam | 0 | 0.0% | 0% |
|  |  | Total | 34 | 100.0% |  |
| **03.2 Skeletal muscle relaxants** | **Injection** | Thiocolchicoside | 8 | 72.7% | 5% |
|  |  | Tolperisone | 2 | 18.2% | 1% |
|  |  | Orphenadrine | 1 | 9.1% | 1% |
|  |  | Baclofen | 0 | 0.0% | 0% |
|  |  | Total | 11 | 100.0% |  |
|  | **Oral** | Tolperisone | 16 | 16.3% | 10% |
|  |  | Tizanidine | 14 | 14.3% | 9% |
|  |  | Thiocolchicoside | 13 | 13.3% | 8% |
|  |  | Cyclobenzaprine | 11 | 11.2% | 7% |
|  |  | Methocarbamol | 6 | 6.1% | 4% |
|  |  | Orphenadrine | 6 | 6.1% | 4% |
|  |  | Orphenadrine + Paracetamol | 6 | 6.1% | 4% |
|  |  | Methocarbamol + Paracetamol | 4 | 4.1% | 3% |
|  |  | Chlorzoxazone | 3 | 3.1% | 2% |
|  |  | Eperisone | 3 | 3.1% | 2% |
|  |  | Baclofen | 2 | 2.0% | 1% |
|  |  | Chlorzoxazone + Paracetamol | 2 | 2.0% | 1% |
|  |  | Mephenoxalone | 2 | 2.0% | 1% |
|  |  | Metaxalone | 2 | 2.0% | 1% |
|  |  | Methocarbamol + Aspirin | 2 | 2.0% | 1% |
|  |  | Thiocolchicoside + Paracetamol | 2 | 2.0% | 1% |
|  |  | Chlorzoxazone + Paracetamol + Diclofenac | 1 | 1.0% | 1% |
|  |  | Cyclobenzaprine + Lysine cloxinate | 1 | 1.0% | 1% |
|  |  | Mefenoxalon | 1 | 1.0% | 1% |
|  |  | Methocarbamol + Ibuprofen | 1 | 1.0% | 1% |
|  |  | Chlorzoxazone + Paracetamol + Aceclofenac | 0 | 0.0% | 0% |
|  |  | Chlorzoxazone + Paracetamol + Ibuprofen | 0 | 0.0% | 0% |
|  |  | Hexafluronium | 0 | 0.0% | 0% |
|  |  | Muscle relaxant (non-descript) | 0 | 0.0% | 0% |
|  |  | Pridinol | 0 | 0.0% | 0% |
|  |  | Sirdalud | 0 | 0.0% | 0% |
|  |  | Total | 98 | 100.0% |  |
|  | **Topical** | Thiocolchicoside | 2 | 100.0% | 1% |
|  |  | Total | 2 | 100.0% |  |
| **10.9 Massage and physical therapy preparations** | **Topical** | Menthol | 27 | 23.1% | 17% |
|  |  | Capsaicin | 25 | 21.4% | 16% |
|  |  | Butane + Propane | 17 | 14.5% | 11% |
|  |  | Methylsalicylate | 17 | 14.5% | 11% |
|  |  | Ethyl chloride | 8 | 6.8% | 5% |
|  |  | Camphor + Menthol | 7 | 6.0% | 4% |
|  |  | Methylsalicylate + Menthol | 5 | 4.3% | 3% |
|  |  | Cold spray | 4 | 3.4% | 3% |
|  |  | Trolamine salicylate | 3 | 2.6% | 2% |
|  |  | Capsaicin + Menthol | 2 | 1.7% | 1% |
|  |  | Camphor | 1 | .9% | 1% |
|  |  | Cold gel | 1 | .9% | 1% |
|  |  | Dimetidine | 0 | 0.0% | 0% |
|  |  | Total | 117 | 100.0% |  |
|  | **Transdermal** | Capsaicin | 1 | 33.3% | 1% |
|  |  | Menthol | 1 | 33.3% | 1% |
|  |  | Methylsalicylate | 1 | 33.3% | 1% |
|  |  | Total | 3 | 100.0% |  |
| **23.1.2 General anaesthetics with analgesia** | **Inhalation** | Methoxyflurane | 10 | 100.0% | 6% |
|  |  | Total | 10 | 100.0% |  |
|  | **Injection** | Ketamine | 1 | 100.0% | 1% |
|  |  | Total | 1 | 100.0% |  |

**Comparison of imported PI medications with the recommended revised OPF**

The importation dataset was used to compare and validate the proposed recommendations for the revised OPF. The graphs below illustrate the prevalence of team importation for the range of medications in each OPF category. The bars of the graphs have been colour coded to visualise the drugs which were recommended for inclusion on the revised OPF through the analysis of the actual-use drug datasets. Green indicates the medications that were recommended for the next OPF.

Figure S1. Prevalence (%) of countries importing various injectable NSAIDs (n=156) [green: listed in revised OPF]

Figure S2. Prevalence (%) of countries importing various oral NSAIDs (n=156) [green: listed in revised OPF]

Figure S3. Prevalence (%) of countries importing various rectal NSAIDs (n=156) [green: listed in revised OPF]

Figure S4. Prevalence (%) of countries importing various topical NSAIDs (n=156) [green: listed in revised OPF]

Figure S5. Prevalence (%) of countries importing various oral COX-2 inhibitor drugs (n=156) [green: listed in revised OPF]

Figure S6. Prevalence (%) of countries importing various transdermal NSAIDs (n=156) [green: listed in revised OPF]

Figure S7. Prevalence (%) of countries importing various injectable opioid drugs (n=156) [green: listed in revised OPF]

Figure S8. Prevalence (%) of countries importing various oral opioid drugs (n=156) [green: listed in revised OPF]

Figure S9. Prevalence (%) of countries importing various medicines for neuropathic pain (n=156) [green: listed in revised OPF]

Figure S10. Prevalence (%) of countries importing various injectable corticosteroids (n=156) [green: listed in revised OPF]

Figure S11. Prevalence (%) of countries importing various injectable local anaesthetics (n=156) [green: listed in revised OPF]

Figure S12. Prevalence (%) of countries importing various topical local anaesthetics (n=156) [green: listed in revised OPF]

Figure S13. Prevalence (%) of countries importing various transdermal local anaesthetics (n=156) [green: listed in revised OPF]

Figure S14. Prevalence (%) of countries importing various oral corticosteroids (n=156) [green: listed in revised OPF]

Figure S15. Prevalence (%) of countries importing various injectable benzodiazepines (n=156) [green: listed in revised OPF]

Figure S16. Prevalence (%) of countries importing various oral benzodiazepines (n=156) [green: listed in revised OPF]

Figure S17. Prevalence (%) of countries importing various injectable skeletal muscle relaxants (n=156)

Figure S18. Prevalence (%) of countries importing various oral skeletal muscle relaxants (n=156) [green: listed in revised OPF]

Figure S19. Prevalence (%) of countries importing various massage and physical therapy preparations (n=156) [green: listed in revised OPF]

**For classes where only one type of drug was imported (not presented by a graph).**

- For injectable COX-2 inhibitors, 3% (n=4) of countries imported parecoxib only.
- For intranasal opioid analgesics, 1% (n=1) of countries imported fentanyl only.
- No countries imported transdermal opioid analgesics.
- For topical skeletal muscle relaxants, 1% (n=2) of countries imported thiocolchicoside.
- For general anaesthetics by inhalation, 6% (n=10) of countries imported methoxyflurane.
- For general anaesthetics by injection, 1% (n=1) of countries imported ketamine.

**Appendix 2****. Results of the team physician survey**

This section presents the survey results of team physicians who participated in either the Tokyo or Beijing Olympic and Paralympic Games. These findings provide an understanding of the perspectives of team physicians in relation to medication use and preferences at these major international sporting events. These results were used to further validate the recommended revised OPF.

**Number of athletes under the care of each team physicians**

Team physicians were asked how many athletes were under their care while at the games. The majority of physicians (67.3%, n=255) reported taking care of 50 or fewer athletes. A smaller proportion (12.4%, n=47) were responsible for 50 to 100 athletes, while 11.9% (n=45) cared for 101 to 200 athletes. Only 8.4% of physicians (n=32) reported that they care for over 200 athletes over the course of the games.

Figure S20. Number of athletes under the care of team physicians (%) at the games

**Clinical specialty of team physicians**

Team physicians were asked what their primary clinical specialty was. The majority of physicians specialised in sports medicine (47.6%, n=182), followed by orthopaedics (18.6%, n=71), general practice primary care (8.6%, n=33), sports medicine surgery (7.9%, n=30), and emergency medicine (3.9%, n=15). There were a mix of 15 other specialties represented by a smaller number of physicians.

Figure S21. Clinical specialty of team physicians (%) working at the games

**Coverage of overall prescribing needs of the OPF**

Team physicians were asked approximately what proportion of their overall prescriptions could be covered by the medicines on the games formulary for the athletes under their care. Of the 381 responding physicians, 76.1% (n=290) stated that most (>75%) or all of their prescribing needs were covered by the formulary. Only 10.2% (n=39) of respondents indicated that less than 25% or none of their prescriptions were covered by the games formulary.

Figure S22. Coverage of overall prescribing needs of formulary versus number of physicians (%)

**Coverage of overall prescribing needs from imported team stock**

Team physicians were asked what proportion of prescriptions for the athletes under their care could be covered by their team’s own stock of medicines. Of the 379 responding physicians, 84.7% (n=321) reported that most (>75%) or all prescriptions could be covered by their own stock. Only 4.7% (n=18) reported that less than 25% of their prescriptions could be covered by their own stock.

Figure S23. Coverage of overall prescribing needs of NOC team stock versus number of physicians (%)

**Expected use of the games pharmacy service**

Team physicians were asked what proportion of the medicines they prescribe for athletes do they expect to be obtained from the polyclinic pharmacy in the athlete village. Of 379 team physician respondents, 19.3% (n=73) reported that most (>75%) or all prescriptions would be obtained from the polyclinic pharmacy. A total of 70.9% (n=269) stated that either few (<25%) or no prescriptions would be expected to be obtained via the polyclinic pharmacy.

Figure S24. Proportion of medicines expected to be obtained from polyclinic pharmacy versus number of physicians (%)

**Expected prescribing of analgesic or anti-inflammatory drugs for athletes**

Team physicians were asked approximately what percentage of athletes under their care do they expect to require analgesic or anti-inflammatory medications for sports-related conditions during the course of the games.

The results show that of the 379 respondents, the majority of physicians (66%, n=250) expected 0-30% of athletes to require analgesic or anti-inflammatory medication during the games. A smaller proportion (21.6%, n=82) expected 31-60% of athletes to require the medication, while even less (12.4%, n=47) expected 61-100% of athletes to require these types of medications.

Figure S25. Percentage groupings of athletes in NOC versus athletes (%) expected to require PI medications for sports-related injury during the games

**Coverage of medications for pain and inflammation on the Olympic & Paralympic medicines formulary**

Team physicians were asked what proportion of their expected PI medications for sports-related conditions could be covered by the medicines in the games formulary. Of 379 physician respondents, 73% (n=277) reported either most (>75%) or all of their PI prescriptions could be covered by the existing formulary. Only 16.7% (n=63) indicated that less than 25% of their prescriptions could be covered.

Figure S26. Proportion of prescribing needs of PI medications covered by the formulary per physician (%)

The next section covers the questions in the survey related to specific medication preferences or frequency of use by team physicians. The drugs recommended for inclusion in the revised OPF are presented as green bars on the graphs below in order to help compare the revised recommended OPF list of medications with the preferences and use of medications by team physicians. This comparison is used to further validate the proposed revised OPF recommendations, as presented in the discussion section that follows.

***Oral medicines***

**Use of oral non-opioid or NSAIDs**

Team physicians were asked which oral non-opioid or NSAIDs they had prescribed in the last 12 months for sports-related conditions in athletes. All physicians reported they had prescribed paracetamol (100%, n=381), with diclofenac (78.2%, n=298) and ibuprofen (77.4%, n=295) also being used by the majority of respondents. Naproxen (42.3%, n=161), celecoxib (36.7%, n=140), meloxicam (25.7%, n=98) was also commonly used. Other drugs were used by less than 25% of physicians in the previous 12 months.

Figure S27. Proportion (%) of team physicians prescribing ORAL non-opioid or NSAIDs in the last 12 months (n=381) [green: listed in revised OPF]

**Use of oral opioid analgesic drugs**

Team physicians were asked which oral opioid analgesic drugs they had prescribed in the previous 12 months to for sports-related conditions in athletes. The most commonly prescribed drugs were oral tramadol (40.8%, n=122) and oral codeine (26.4%, n=79). Other drugs in this class were prescribed by less than 6% of physicians.

Figure S28. Proportion (%) of team physicians prescribing ORAL opioid analgesic drugs in the last 12 months (n=381) [green: listed in revised OPF]

**Oral medicines for mild to moderate pain without inflammation**

Team physicians were asked which oral medicine they most frequently prescribe for mild to moderate pain (without inflammation) for sports-related conditions in athletes. The most commonly prescribed drugs included paracetamol (85.3%, n=324), ibuprofen (43.2%, n=164), diclofenac (31.3%, n=119) and naproxen (16.1%, n=61). Other drugs for this clinical indication were used by less than 10% of physicians.

Figure S29. Proportion (%) of team physicians reporting their most frequently prescribed ORAL medicine for mild to moderate pain (without inflammation) for sports-related conditions in athletes (n=380) [green: listed in revised OPF]

**Oral medicine for moderate to severe pain without inflammation**

Team physicians were asked which oral medicine they most frequently prescribed for moderate to severe pain (without inflammation) for sports-related conditions in athletes. The most common prescribed drugs were paracetamol (44.7%, n=170), diclofenac (36.1%, n=137), tramadol (32.6%, n=124), codeine + paracetamol as a combination formulation (30.8%, n=117) and ibuprofen (30.5%, n=116). Other drugs were prescribed by less than 15% of physicians for this clinical indication.

Figure S30 Proportion (%) of team physicians reporting their most frequently prescribed ORAL medicine for moderate to severe pain (without inflammation) for sports-related conditions in athletes (n=380) [green: listed in revised OPF]

**Oral medicine for mild to moderate pain with inflammation**

Team physicians were asked which oral medicine they most frequently prescribe for mild to moderate pain with inflammation for sports-related conditions in athletes. The most commonly prescribed drugs included ibuprofen (55.6%, n=212), diclofenac (55.4%, n=211), paracetamol (40.2%, n=153), naproxen (24.4%, n=93), celecoxib (19.2%, n=73), and ketoprofen (15.2%, n=58). Other drugs were prescribed by less than 15% of physicians.

Figure S31. Proportion (%) of team physicians reporting their most frequently prescribed ORAL medicine for mild to moderate pain with inflammation for sports-related conditions in athletes (n=381) [green: listed in revised OPF]

**Oral medicine for moderate to severe pain with inflammation**

Team physicians were asked which oral medicine they most frequently prescribe for moderate to severe pain with inflammation for sports-related conditions in athletes. The most commonly prescribed medicines included diclofenac (54.9%, n=208), ibuprofen (38.0%, n=144), paracetamol (31.4%, n=119), tramadol (25.1%, n=95), ketoprofen (22.4%, n=85), celecoxib (22.4%, n=85), naproxen (21.6%, n=82), codeine + paracetamol (21.4%, n=81) and etoricoxib (18.5%, n=70). Other drugs were prescribed by less than 15% of physicians.

Figure S32. Proportion (%) of team physicians reporting their most frequently prescribed ORAL medicine for moderate to severe pain with inflammation for sports-related conditions in athletes (n=379) [green: listed in revised OPF]

**Oral medicines for neuropathic pain**

Team physicians were asked which oral medicine they would most frequently prescribe for neuropathic pain in athletes. The most commonly prescribed drugs included gabapentin (46.6%, n=172), pregabalin (45.0%, n=166), diclofenac (25.2%, n=93), tramadol (21.4%, n=79), and amitriptyline (21.1%, n=78). Other drugs were prescribed by less than 14% of physicians for the treatment of neuropathic pain.

Figure S33. Proportion (%) of team physicians reporting their most frequently prescribed ORAL medicine for neuropathic pain in athletes (n=369) [green: listed in revised OPF]

**Oral skeletal muscle relaxants**

Team physicians were asked which skeletal muscle relaxants they had prescribed in the last 12 months for sports-related conditions in athletes. The most commonly prescribed medications were diazepam (25.0%, n=70), cyclobenzaprine (21.8%, n=61), baclofen (9.6%, n=55), and tizanidine (18.2%, n=51). Other drugs in this category were prescribed by less than 14% of responding physicians.

Figure S34. Proportion (%) of team physicians reporting which skeletal muscle relaxants they had prescribed in the last 12 months for sports-related conditions in athletes (n=280) [green: listed in revised OPF]

***Injectable medicines***

**Injectable non-opioid and non-steroidal anti-inflammatory drugs (NSAIDs)**

Team physicians were asked which injectable non-opioid and non-steroidal anti-inflammatory drugs (NSAIDs) they had prescribed in the last 12 months for sports-related conditions in athletes. The most commonly prescribed medications were diclofenac (48.7%, n=148), ketorolac (23.0%, n=70) and ketoprofen (15.5%, n=47). Other injectable drugs in these categories were prescribed by less than 15% of physicians.

Figure S35. Proportion (%) of team physicians reporting which INJECTABLE non-opioid and non-steroidal anti-inflammatory drugs (NSAIDs) they had prescribed in the last 12 months for sports-related conditions in athletes (n=304) [green: listed in revised OPF]

**Injectable opioid analgesic drugs**

Team physicians were asked which injectable opioid analgesic drugs they had prescribed in the last 12 months for sports-related conditions in athletes. Tramadol was the most prescribed injectable opioid analgesic drug (18.3%, n=46), followed by much lower prescribing of morphine (3.6%, n=9) and fentanyl (3.6%, n=9), and even lower prescribing of other drugs in this class.

Figure S36. Proportion (%) of team physicians reporting which INJECTABLE opioid analgesic drugs they had prescribed in the last 12 months for sports-related conditions in athletes (n=251) [green: listed in revised OPF]

**Injectable medicine for moderate to severe pain without inflammation**

Team physicians were asked which injectable medicine they most frequently prescribe to athletes for moderate to severe pain without inflammation due to sports-related conditions. The most commonly prescribed drugs included diclofenac (28.1%, n=99), paracetamol (19.0%, n=67), ketorolac (17.0%, n=60) and tramadol (16.5%, n=58). Other injectable drugs for this clinical indication were prescribed by less than 15% of responding physicians.

Figure S37. Proportion (%) of team physicians reporting which INJECTABLE medicine they most frequently prescribe to athletes for moderate to severe pain without inflammation due to sports-related conditions (n=352) [green: listed in revised OPF]

**Injectable medicine for moderate to severe pain with inflammation**

Team physicians were asked which injectable medicine they most frequently prescribe to athletes for moderate to severe pain with inflammation due to sports-related conditions. The two most commonly prescribed drugs by far were diclofenac (41.4%, n=149) and ketorolac (20.3%, n=73). Other injectable drugs for this clinical indication were prescribed by less than 14% of physicians.

Figure S38. Proportion (%) of team physicians reporting which INJECTABLE medicine they most frequently prescribe to athletes for moderate to severe pain with inflammation due to sports-related conditions (n=360) [green: listed in revised OPF]

**Glucocorticoid medicines for local injection**

Team physicians were asked which glucocorticoid medications they most frequently administer to athletes by local injection (e.g. intra-articular) for inflammation due to sports injury. The four most prescribed drugs include triamcinolone (36.8%, n=131), betamethasone (22.2%, n=79), dexamethasone (21.1%, n=75) and methylprednisolone (20.8%, n=74). Other injectable glucocorticoids for local administration were prescribed by less than 10% of physicians.

Figure S39. Proportion (%) of team physicians reporting which glucocorticoid medications they most frequently administer to athletes by local injection (e.g., intra-articular) for inflammation due to sports injury (n=356) [green: listed in revised OPF]

**Local anaesthetic for local injection into a joint**

Team physicians were asked which local anaesthetic drug they most frequently administer to athletes by local injection into a joint (e.g. intra-articular) for pain due to sports injury. The most commonly prescribed drug by far was lidocaine (70.1%, n=255), followed by bupivacaine (23.1%, n=84). Other local anaesthetic drugs for local injection were prescribed by less than 10% of team physicians.

Figure S40. Proportion (%) of team physicians reporting which local anaesthetic drug they most frequently administer to athletes by local injection into a joint (e.g., intra-articular) for pain due to sports injury (n=364) [green: listed in revised OPF]

**Local anaesthetic for local injection into soft tissue**

Team physicians were asked which local anaesthetic drug they most frequently administer to athletes by local injection into soft tissue for sports-related conditions. The most commonly prescribed drug by far was lidocaine (74.0%, n=268), followed by bupivacaine (23.2%, n=84). Other drugs were prescribed by less than 7% of physician respondents for this indication.

Figure S41. Proportion (%) of team physicians reporting which local anaesthetic drug they most frequently administer to athletes by local injection into soft tissue for sports-related conditions (n=362) [green: listed in revised OPF]

***Urgent or emergency treatment***

**Emergency analgesia for severe pain on the field of play**

Team physicians were asked which medicine they prefer to use for emergency analgesia for severe pain on the field of play due to serious injury in athletes. The most commonly preferred drugs were tramadol injection (36.6%, n=132), morphine injection (20.2%, n=73), fentanyl injection (15.8%, n=57), ketamine injection (11.4%, n=41) and methoxyflurane for inhalation (11.1%, n=40). Other drugs for this use were preferred by less than 8% of physicians.

Figure S42. Proportion (%) of team physicians reporting which medicine they prefer to use for emergency analgesia for severe pain on the field of play due to serious injury in athletes (n=361) [green: listed in revised OPF]

**Analgesia with sedation requiring rapid onset on the field of play**

Team physicians were asked which medication they prefer to use for analgesia with sedation requiring rapid onset on the field of play. The most commonly preferred drugs were fentanyl (22.8%, n=81), tramadol (19.4%, n=69), morphine (16.9%, n=60) and ketamine (15.4%, n=55). Other drugs for this use were preferred by less than 10% of physicians.

Figure S43. Proportion (%) of team physicians reporting which medication they prefer to use for analgesia with sedation requiring rapid onset on the field of play (n=356) [green: listed in revised OPF]

***Topical medicines***

**Topical medicines for pain and inflammation**

Team physicians were asked which topical medicine they most frequently prescribe for pain and inflammation due to sports-related conditions. The three most commonly prescribed drugs were NSAIDs including diclofenac (78.2%, n=297), ibuprofen (33.4%, n=127) and ketoprofen (27.1%, n=103). Other drugs included lidocaine (23.2%, n=88), menthol (21.1%, n=80) and capsaicin (15.8%, n=60). Other topical medicines for pain and inflammation were prescribed by less than 12% of responding physicians.

Figure S44. Proportion (%) of team physicians reporting which topical medicine they most frequently prescribe for pain and inflammation due to sports-related conditions (n=380) [green: listed in revised OPF]

**Potential for follow-up studies with the sample population**

Of all respondents, 77.2% (n=295) agreed that they would be happy to be contacted in the future to participate in follow-up studies for this project or similar research.
